# Safety of Elixinol Hemp Extract: *In Vitro* Genetic Toxicity and Subchronic Toxicity in Rats

**DOI:** 10.1155/2023/5982883

**Published:** 2023-12-11

**Authors:** Margitta Dziwenka, Laurie C. Dolan, Mithila Rao

**Affiliations:** ^1^GRAS Associates, LLC 11810 Grand Park Avenue, Suite 500, North Bethesda, MD 20852, USA; ^2^Product Safety Labs, 2394 US Highway 130, Dayton, NJ 08810, USA

## Abstract

The results of safety studies performed with Elixinol Hemp Extract, a blend of hemp extract, cannabidiol (CBD) isolate, and copaiba containing approximately 65% total CBD, are described in this paper. In a 15-day range-finding study in rats, there were no effects of treatment with up to 101.4 mg/kg bw/day of the extract by gavage on any safety parameter measured in the study, with the exception that centrilobular hepatocellular hypertrophy occurred in all treatment groups, which correlated with increases in absolute liver weight in high-dose females and liver to terminal body weight ratio in mid-dose and high-dose females. A GLP-compliant 90-day OECD Guideline 408 study in rats that included a behavioral battery and a 28-day recovery phase was also conducted with Elixinol Hemp Extract administered by gavage. The doses used in the 90-day study were 0 (vehicle), 28.94, 50.64, and 86.81 mg/kg bw/day. The findings were similar to those observed in the range-finding study. There were no effects of the test material on any test parameter in the 90-day study other than findings related to the liver (increased liver weight in high-dose main study males and mid-dose and high-dose main study females and low incidences of hepatocellular hypertrophy and vacuolation in main study high-dose males). Similar findings were not observed in the recovery animals, and there were no alterations in the clinical chemistry suggestive of liver toxicity in any of the main study or recovery animals. Therefore, the liver outcomes observed in the main study were not considered adverse. The test material also tested negative for mutagenicity in bacterial reverse mutation assays (plate incorporation and preincubation) in the absence and presence of metabolic activation. The results indicate that the oral 90-day no observed adverse effect level (NOAEL) of Elixinol Hemp Extract in rats is 86.81 mg/kg bw/day (highest dose administered), and that the extract is not mutagenic.

## 1. Introduction

In December 2018, with the passage of the Agriculture Improvement Act of 2018 (also referred to as the 2018 Farm Bill), “hemp” was defined as the *Cannabis sativa* L. plant with a “tetrahydrocannabinol (THC) concentration of not more than 0.3 percent on a dry weight basis” [1]. In turn, the act removed hemp from the definition of marijuana in the controlled substances act and from schedule I. Importantly, the 2018 Farm Bill permits interstate commerce of hemp-derived products for commercial or other purposes. This act created a boom in the market for hemp products, especially those containing cannabidiol (CBD), one of the main phytocannabinoids in the *Cannabis sativa* L. plant.

In June 2018, the FDA approved Epidiolex®, a formulation of purified cannabis-derived CBD, as an antiseizure medication for use in patients with Dravet and Lennox–Gastaut syndromes [2]. Safety information supplied to the FDA for this approval included results from a number of different preclinical and clinical studies. Although the FDA approved Epidiolex® for a specific indication, they recognized that risks were associated with the use of the drug, particularly at high concentrations. Results of studies with Epidiolex® are applicable for CBD isolates but not for hemp extracts such as Elixinol, which are typically complex mixtures containing substances that could influence the pharmacological and/or toxicological profile of CBD. In support of this hypothesis, a recent meta-analysis indicated that a lower dosage of a CBD-containing hemp extract was needed to manage symptoms related to refractory epilepsy compared to the amount of purified or isolated CBD preparations needed for the same benefit (6.0 mg/kg/day in CBD-rich plant extracts versus 25.3 mg/kg/day in purified CBD users) [3]. Fewer side effects were also noted in subjects taking the extract versus the purified CBD preparation, suggesting that other substances in the extract mitigate the potential for CBD to cause adverse effects.

Hemp extracts may contain more than 100 different cannabinoids, flavonoids, terpenes, and numerous other phytochemicals [4]. Similar to other botanical extracts, the levels of different phytochemicals in hemp extracts may differ between cultivars or methods of manufacture. This can influence bioactivity and the potential for the extracts to cause toxicity. Environmental or process-induced contaminants or diluents used in various preparations may also influence study and safety outcomes. Therefore, it is important to determine the safety of hemp extracts on an individual basis.

To date, safety studies performed on CBD-containing hemp extracts include *in vitro* genetic and 90-day studies in rats with hemp extracts containing approximately 6% or 25% CBD (diluted in olive oil and sunflower oil, respectively), and a carcinogenicity study in rats with an extract containing 64.6–67.2% CBD [5–7]. In the 90-day studies, no observed adverse effect levels (NOAELs) obtained for the extracts were 400 and 800 mg/kg bw per day for males and females, respectively [5] and 100 mg/kg bw/day for males and 360 mg/kg bw/day for females, respectively [6], compared to a 26-week NOAEL in male and female rats of 150 mg/kg bw per day for Epidiolex® [7]. The relatively high NOAEL for the product diluted in olive oil compared to the other products is likely due, in part, to the dilution of the hemp extract in a high amount of olive oil (91%).

Three studies were performed to assess the safety of Elixinol Hemp Extract including a reverse mutation (Ames) test to assess the ability of the extract to cause genetic toxicity, a repeated dose, a range-finding study in rats to determine doses to use in a longer-term study, and a 90-day repeated dose study in rats to determine the NOAEL.

## 2. Materials and Methods

### 2.1. Test Articles

The test product (Elixinol Hemp Extract) was an herbal blend produced using supercritical CO_2_ extraction and containing approximately 65% of total CBD that was comprised of approximately 62% of hemp extract, 8% of CBD isolate, and 30% of copaiba. The source was Elixinol, Westminster, CO 80021. For the reverse mutation studies, the test product was formulated as a solution in dimethylsulfoxide (DMSO) to provide dose levels of CBD of up to 5000 *μ*g/plate. The solutions were vortexed prior to use. The positive control chemicals sodium azide, ICR 191 acridine, daunomycin, methylmethanesulfonate (MMS), and 2-aminoanthracene, as well as the bacterial strains and the S9 (9000×g) tissue fraction used in the reverse mutation study, were sourced from Molecular Toxicology, Inc. The S9 tissue fraction used in the reverse mutation study was isolated from livers of Sprague–Dawley rats induced with phenobarbital and 5,6-benzoflavone. The S9 fraction of rats contains free endoplasmic reticulum, microsomes, cytosolic enzymes, and some cofactors and is typically used in *in vitro* genetic toxicity studies to metabolize test articles.

The vehicle used for the range-finding and 90-day studies in rats was medium-chain triglyceride (MCT) oil (Connoils LLC, WI, USA). The formulation was placed in a heated water bath until it reached a temperature between 48.9 and 65.6°C for the range-finding study and 53.6–56.6°C for the 90-day study, and the formulation was mixed until it appeared homogenous. A stock solution was prepared by mixing test material and vehicle thoroughly at room temperature. Dosing formulations were prepared from the stock solution weekly, after mixing the solution until it appeared to be homogenous. Prior to daily dosing, the solutions were stirred until homogenous and cooled to physiological temperature.

Dosing formulations for the range-finding and 90-day studies were tested by a validated high-performance liquid chromatography (HPLC) method for stability over the course of the study, homogeneity, and concentration.

### 2.2. Guidelines

The bacterial reverse mutation and the 90-day toxicity studies were conducted in compliance with Good Laboratory Practices as stated in U.S. FDA GLP: 21 CFR Part 58, 1987, with the exception that characterization of the positive control substances and verification of the concentration of the positive control substances in their carriers during the bacterial reverse mutation study were not determined analytically. The bacterial reverse mutation studies were performed according to the following guidelines: U.S. FDA Toxicological Principles for the Safety Assessment of Food Ingredients (Redbook) IV.C. 1. a. (2007) and ICH S2 (R1) Guidance on Genotoxicity Testing and Data Interpretation for Pharmaceuticals Intended for Human Use (2012). The range-finding study in rats was conducted according to OECD Guideline 407 and Redbook IV.C. 3. a. (2007), and the 90-day study in rats was conducted according to OECD Guideline 408 and Redbook IV.C. 4. a. (2007). The reverse mutation studies and in-life procedures and tissue harvests were performed at the Product Safety Labs' (PSL) test facility in Dayton, New Jersey, which is AAALAC (Association for Assessment and Accreditation of Laboratory Animal Care) accredited. All procedures involving the use of animals in the described studies were thoroughly reviewed and unanimously approved by the PSL Institutional Animal Care and Use Committee under Animal Use Protocols P710 and P713 on September 17, 2019. Histopathology of tissues from the range-finding and 90-day studies was performed at Histo-Scientific Research Laboratories (HSRL), Mount Jackson, VA.

### 2.3. Bacterial Reverse Mutation Assay

The potential for the test material to cause mutations was examined in bacterial reverse mutation assays using a plate incorporation method and a preincubation method as described by Dolan et al. [8] using *S. typhimurium* strains TA98, TA100, TA1535, and TA1537 and *E. coli* WP2uvrA in the presence and absence of an S9 metabolizing system. Three replicate plates were used at each test point, and appropriate sterility control check plates (treated with critical components in the absence of bacteria) were included.

For the plate incorporation method (experiment 1), plates were prepared as previously described [8] by using an overlay agar supplemented with biotin and limited amounts of histidine and tryptophan (obtained from Molecular Toxicology, Inc.) poured over the surface of a minimal glucose agar plate (obtained from Molecular Toxicology, Inc.). For the preincubation method (experiment 2), all substances except for the overlay agar were mixed together and incubated for 30 min at 37°C prior to mixing with the overlay agar and pouring onto the minimal agar plates. Plates were inverted after solidification and incubated at 37°C for approximately 65 hours prior to counting.

The concentrations of test articles used in both experiments were 2.286, 7.234, 22.86, 72.34, 228.6, 723.4, 2286, and 7234 *μ*g/plate. The positive controls in the absence of the S9 mix were daunomycin for *S. typhimurium TA98*, sodium azide for TA100 and TA1535, ICR 191 acridine for TA1537, and MMS for *E. coli* WP2uvrA. The positive control for all bacterial strains in the presence of the S9 mix was 2-aminoanthracene (2-AA). DMSO served as the negative control. After incubation, the number of colonies per plate was counted manually and/or with the aid of a plate counter (Colony Plate Reader: Model ColonyDoc-ItTM). The mean and standard deviation were calculated for each set of triplicate plates. Toxicity was assessed by observing a substantial dose-related reduction in revertant colony counts compared with lower dose levels and the concurrent vehicle control or a thinning of the background lawn.

The assay was considered valid if mean plate counts for untreated and positive control plates were within the expected range taking into account the laboratory historical control range and/or published values, the background lawn for vehicle control plates appeared normal, and positive controls produced substantial increases in revertant colony numbers. The test substance was considered mutagenic if at least twofold increases in mean revertant numbers were observed for strains TA98, TA100, and WP2uvrA and threefold increases for strains TA1535 and TA1537, with mean values outside the laboratory historical control range. The increases must occur at more than one experimental point (at least one strain, more than one dose level, more than one occasion, or with different methodologies). A test substance that produces neither a concentration-related increase in the number of revertant colonies nor a reproducible substantial increase in revertant colonies is considered to be nonmutagenic in the test system.

### 2.4. Range-Finding and 90-Day Studies

Rats used in the range-finding and 90-day studies were CRL Sprague–Dawley CD® IGC rats (Charles River Laboratories, Inc., Raleigh, NC). The animals were acclimated for seven days before the start of the studies and were seven to eight weeks old at study initiation. Animals were housed and selected for use in the study as previously described [8]. The temperature and relative humidity of the animal room were as follows: 20–24°C and 42–76% for the range-finding study and 18–23°C and 35–68% for the 90-day study. Rats were fed a basal diet (2016 Certified Teklad Rodent Diet® from Envigo Teklad, Inc.) and provided filtered tap water *ad libitum* throughout the study. The day before study termination food was withdrawn.

For the range-finding study, 40 rats (20/sex) were assigned to four treatment groups (5/sex/group): vehicle control, 43.5 mg/kg bw/day, 72.5 mg/kg bw/day, and 101.4 mg/kg bw/day Elixinol Hemp Extract, by gavage. The highest dose chosen for the range-finding study was not expected to cause marked toxicity based on previous studies performed with hemp extracts and/or CBD, and the intermediate-dose and low-dose levels were selected to derive a dose-response for any effects observed. Individual doses were calculated based on the most recent weekly body weights and were adjusted each week to maintain the targeted dose level for all rats (i.e., mg/kg/day). All doses were administered volumetrically at 5 mL/kg. The control group received the vehicle only, at the same dose volume as the test animals.

For the range-finding study, body weights were recorded prior to the start of the study, weekly throughout the study, and immediately before study termination (day 16). Body weight gain, food consumption, and food efficiency were determined, and clinical observations were recorded. The animals were observed at least twice a day for mortality and once per day for signs of gross toxicity. On day 16 (after an overnight fast), blood samples were collected from the inferior vena cava under isoflurane anesthesia at termination. Blood samples were spun in a refrigerated centrifuge to obtain serum. Serum samples were stored at −80°C until analysis for clinical chemistry parameters as recommended by the Guidelines stated above. All animals were subjected to gross necropsy. The liver, kidneys (combined), and adrenal glands (combined) of animals were weighed and preserved in 10% neutral buffered formalin. Tissues from control and high-dose animals were processed, embedded in paraffin, sectioned, stained with hematoxylin and eosin (H&E), and examined by light spectroscopy. In addition, based on a target organ identified during slide evaluation, sections of the liver from all animals in the low-dose and middle-dose groups were also prepared and examined by light spectroscopy.

For the 90-day study, 120 rats (60/sex) were assigned to four study treatment groups (15/sex/group): vehicle control, 28.94 mg/kg bw/day, 50.64 mg/kg bw/day, and 86.81 mg/kg bw/day Elixinol Hemp Extract and were dosed in the same manner as the range-finding study. The corresponding amounts of CBD are 0, 18.95, 33.16, and 56.84 mg/kg bw/day, respectively. The doses for the 90-day study were chosen based on the results of the range-finding study. Ten animals/sex per group served as main study animals and 5/sex/group were maintained on the study for an additional 28 days without any treatment and served as recovery animals. For the 90-day studies, body weights were recorded prior to the start of the study, weekly throughout the study, and immediately before study termination (day 16 for the range-finding study, days 93 (males) and 94 (females) for the 90-day study, and day 121 for recovery animals from the 90-day study). In-life parameters measured in the 90-day study were the same as those determined for the range-finding study. In addition, ophthalmic examinations (conducted during the acclimation period and on day 88), functional observational battery (FOB) (during week 12), and urinalysis (during week 13) were conducted as previously described [8].

At the termination of the 90-day study, blood samples for clinical chemistry were collected as described for the range-finding study. Serum was prepared, stored, and analyzed for the same parameters as measured for the range-finding study plus for triiodothyronine (T3), thyroxine (T4), thyroid stimulating hormone (TSH), high-density lipoprotein (HDL), and low-density lipoprotein (LDL). Standard hematological/coagulation parameters were also measured in blood collected on an anticoagulant (K_2_EDTA), as recommended in the guidelines stated above. In addition, separate blood smears were prepared from each animal undergoing hematological evaluation and if necessary, were stained with Wright–Giemsa stain and examined to substantiate or clarify the results of the hematology findings. All blood analyses were performed on samples from the main study and recovery animals, with the exception of the thyroid hormone analyses, which were only performed on main study animals.

At scheduled termination, all animals in the 90-day study were euthanized under anesthesia and subjected to a full necropsy. Organs were collected, weighed, and preserved for histopathological analysis as previously described [8]. Histopathological examinations were carried out on the organs and tissues of all animals in the control and high-dose groups, as well as on organs with gross lesions. The examinations were performed by a board-certified veterinary pathologist (DAVCP). At the discretion of the pathologist, examinations were also conducted on tissues and organs from the low-dose and middle-dose groups to further investigate if any lesions discovered in the high-dose animals also occurred at lower doses.

### 2.5. Statistical Analyses for the Range-Finding and 90-Day Studies

Statistical analysis was carried out as previously described [8], with the following modifications. Statistical analysis was conducted by using one or more of the following software applications: Provantis® version 9, Tables and Statistics, Instem LSS, Staffordshire, UK; Pristima® version 7, Reporting, Xybion Corporation, Lawrenceville, NJ; or Prism Biostatistics, GraphPad Software, San Diego, CA. Normally distributed (tested using Bartlett's or Levene's test) and homogenous data (tested using Shapiro–Wilk's test) were analyzed using a two-way (continuous data.) or one-way analysis of variance (ANOVA). Comparisons of the treated groups to control were carried out using Dunnett's *t*-test for multiple comparisons. Data that did not pass the normal distribution or homogeneity tests were analyzed by a Kruskal–Wallis nonparametric ANOVA, and a Dunn's test. Data were evaluated at the *p* < 0.05, *p* < 0.01, and *p* < 0.001 levels of significance, with *p* < 0.05 chosen as the minimum criterion for statistical significance.

## 3. Results

There were no test substance-related increases in the number of revertant colonies for *S. typhimurium* strains TA98, TA100, TA1535, TA1537 or *E. coli* WP2 uvrA in both the absence and presence of S9 using either the plate incorporation or the preincubation method (Supplementary Table 1). For all strains, at least five nontoxic dose levels without precipitate were evaluated and therefore bacterial mutagenicity was adequately assessed. Toxicity with evidence of incomplete lawn was noted for TA100 at 723.4 *μ*g/plate without S9 in the preincubation method and precipitate which obstructed lawn evaluation was observed for TA98, TA100, TA1535, and TA1537 at doses ≥2286 *μ*g/plate ± S9 in both experiments and for *E. coli* at dose level ≥2286 *μ*g/plate ± S9 in experiment 1 only. The assay met all three criteria for validity for each strain and was therefore considered valid.

For the range-finding study, the results of the stability analysis were 103.7% on day 1 (initial) and 101.6% on day 15 (final) of the target concentration. The difference in the neat test substance concentration over the course of the study was −2.1%, and the overall test substance stability was determined to be 97.9%. Representative samples of the low-dose and high-dose concentrations taken from the top, middle, and bottom of the dose preparations on day 1 were analyzed. Homogeneity analysis resulted in a relative standard deviation (RSD) of 0.3% for the samples. The average nominal concentration of the dose preparations on day 1 was 116.8 and 120.1% of the target concentrations of 8.7 and 20.3 mg/mL, respectively.

No deaths or macroscopic observations were observed in the range-finding study. There were no effects of the test material on body weight gain, food consumption or food efficiency, and no test material-related clinical observations or changes in serum clinical chemistry. Centrilobular hepatocellular hypertrophy was observed in all treated animals, with average severity increasing with dose. The finding correlated with statistically significant increases in liver weight in high-dose females (*p* < 0.05) and liver-to-terminal body weight ratio in mid-dose and high-dose females (*p* < 0.01). The results indicated that rats would be expected to tolerate up to 101.4 mg/kg bw/day of the test material in a longer-term study.

For the 90-day study, the results of the stability analysis were 100.4% of the target concentration on day 0 (initial), 101.2% on day 49 (middle), and 99.0% on day 91 (final). The difference in the neat test substance concentration over the course of the study was of −1.4%, and the overall test substance stability was determined to be 98.6%. Homogeneity analysis of the dose preparations resulted in an RSD of 1.2, 0.4, and 0.9% for concentrations of 5.79, 10.13, and 17.36 mg/mL, respectively. Concentration verification analysis samples were collected on the day of initial (day 0, as part of the homogeneity assessment), middle (day 49), and final (day 91) preparations. The day 0 samples averaged 117.6, 113.8, and 113.2%, the day 49 samples averaged 111.3, 112.9, and 112.9%, and the day 91 samples averaged 109.9, 108.2, and 109.7% of the target concentrations of 5.79, 10.13, and 17.36 mg/mL, respectively. Concentration verification results were considered to have met the target concentrations within an acceptable margin of variation.

**Figure 1:**
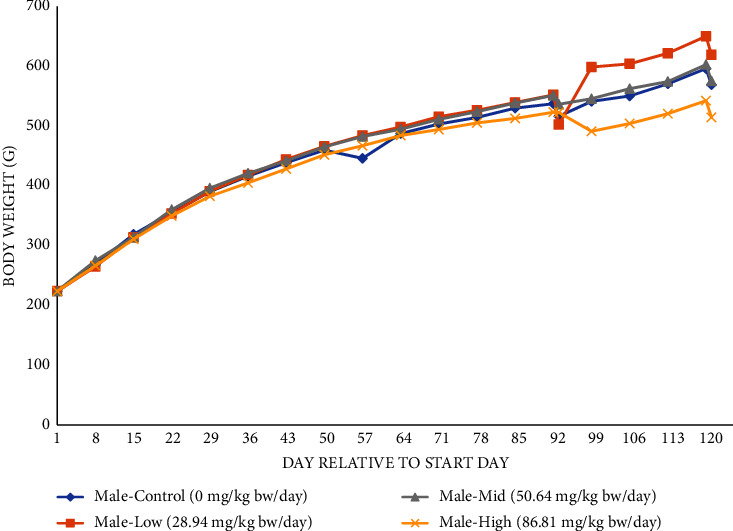
Body weights for the 90-day study of the main study and recovery animals—males^*∗*^. ^*∗*^Data are for 15 animals/group from days 1 to 91 (includes main study and recovery animals), 10 animals/group on day 92 (main study animals only, at termination), and 5 animals/group from days 99 to 121 (recovery animals only).

**Figure 2:**
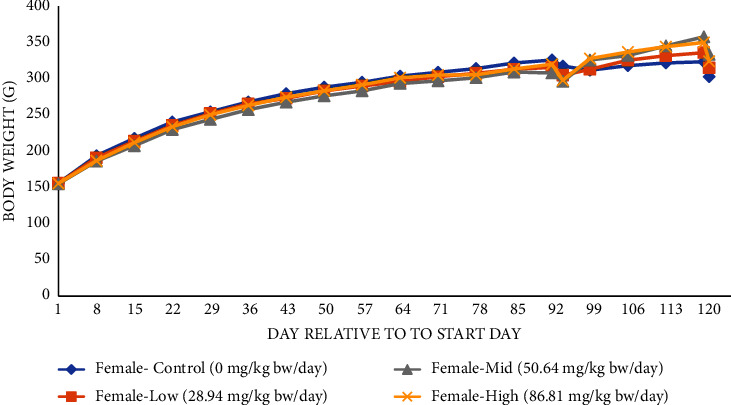
Body weights for the 90-day study of the main study and recovery animals—females^*∗*^. ^*∗*^Data are for 15 animals/group from days 1 to 91 (includes main study and recovery animals), 10 animals/group on day 92 (main study animals only, at termination), and 5 animals/group from days 99 to 121 (recovery animals only).

There were no mortalities throughout the duration of the 90-day study (including the recovery period), and there were no test substance-related clinical or FOB observations. Ophthalmologic examinations of all animals were normal. A palpable, firm round mass was found in the left hindlimb region of one mid-dose female during the recovery period, which was measured to be of 30 mm × 25 mm × 12 mm on day 102 and gradually increased in size to 50 mm × 40 mm × 20 mm by day 120. This mass was considered incidental and not treatment-related. Mean body weights (Figures 1 and 2) and mean daily body weight gain of animals (Supplementary Table 2) receiving the test article were not statistically different from controls throughout the study (including the recovery period). Although the mean body weight of high-dose males appeared to be lower than controls towards the end of the study, they were within 10% of the control group. The slightly lower weights of high-dose males corresponded with slightly lower food consumption at a few time points observed with this group, including during the recovery period (Supplementary Table 3). The changes in weight appear in Figures 1 and 2. 91–93 are artifacts of change reporting over from all animals to recovery animals and are not related to the test material. The decreases in body weight from day 120 to 121 are likely due to overnight fasting. Food consumption in all treated female groups was higher than that in controls during the recovery period, but this was not dose-dependent and had no effect on body weight. There was no effect of the test material on the food efficiency of males or females during the entire study, with the exception of a sporadic increase in food efficiency of high-dose males from days 57 to 64.

There were no changes in hematology between groups, with the exception of some statistically significant differences in some parameters in mid-dose and high-dose females in the recovery groups (decreased NEU at the mid-dose and increased NEU at the high-dose (*p* < 0.01), decreased LUC at the mid-dose (*p* < 0.05), and increased RET at the mid-dose (*p* < 0.05), none of which were attributed to the test material because there was no effect on these parameters in main study animals (Supplementary Table 4). Statistically significant changes in clinical chemistry parameters were observed in some groups, as follows: decreased BILI in high-dose males, decreased sodium in mid-dose males, increased HDL and ALB in all groups of treated females, increased ALB in low-dose recovery group females, and decreased TSH in mid-dose and high-dose females. The only changes in clinical chemistry in the main study or recovery group animals given the Elixinol Hemp Extract that appeared to be dose-dependent, were a decrease in BILI that was statistically significant from control in high-dose males, increased serum albumin in all groups of treated females and in low-dose recovery females, and decreased TSH in mid-dose and high-dose females (Table 1). The toxicological relevance of these data is discussed in the Discussion section. There were no statistically significant differences between the urinalysis parameters of treated animals and control animals.

Liver weights of treated male and female rats increased in a dose-dependent manner (Table 2). They were statistically significant from control for high-dose main study males if expressed in terms of absolute weight or weight relative to body weight or brain weight (*p* < 0.01 − 0.001). Statistically significant increases (*p* < 0.01 − 0.001) in liver weight were observed in mid-dose and high-dose females when expressed in terms of body weight. The increases in liver weight were reversible, as they were not observed in recovery animals.

There were no macroscopic observations attributable to the administration of Elixinol Hemp Extract. Three low-dose and one mid-dose female rats contained fluid-filled uteri, which were consistent with physiologic changes secondary to the estrus cycle. One mid-dose male contained a red, left mandibular lymph node, which was not correlated microscopically, and another mid-dose male had a mottled thymus, which consisted of minimal congestion microscopically. One high-dose female had a small left adrenal, which was attributable to a necropsy collection artifact; the capsule was missing from this gland, and the tissue was otherwise normal upon microscopic examination. One high-dose male animal had several urogenital abnormalities that stemmed from urinary bladder calculi as well as moderate diffuse granulocytic hyperplasia of the bone marrow. This animal's prostate was described macroscopically as containing an 11 × 8 × 5 mm mass, which consisted microscopically of moderate multifocal chronic-active inflammation secondary to the urinary bladder findings. The changes in this animal were not attributed to the test material due to the lack of similar findings in other animals.

Histopathological examinations were performed on the liver and pancreas from all males in the main study and recovery groups, as well as other organs from control and high-dose animals. The results are shown in Supplementary Table 5. Changes in the pancreas were considered unrelated to test material administration due to low incidence and lack of a dose-response relationship. Minimal hepatocellular hypertrophy was observed in 2 out of 10 males in both the mid-dose and high-dose main study groups; one of the high-dose males also exhibited hepatocellular vacuolation. All other microscopic observations noted in the main study animals were considered to be incidental by the study pathologist. There were no microscopic alterations in treated female animals that were attributed to test material administration. No liver findings were noted in treated males after the 28-day recovery period, indicating reversibility. There were also no corresponding alterations in clinical chemistry, therefore the liver findings in a few main studies of males were not considered adverse.

## 4. Discussion

The studies reported in this manuscript were conducted to evaluate the toxicological profile of Elixinol Hemp Extract, a unique combination of hemp extract, CBD, and copaiba oil. A bacterial reverse mutation assay was conducted at levels of up to 7234 *µ*g Elixinol Hemp Extract/plate in both a plate incorporation and preincubation assay. The results of these assays demonstrate that the extract is nonmutagenic in this test system, while the results from the oral range-finding study indicate that Sprague–Dawley rats would be able to tolerate doses of up to 101.4 mg/kg bw/day in a study of longer duration.

In the 90-day repeat dose study conducted with doses of 28.94, 50.64, and 86.81 mg/kg bw/day, no mortalities were reported, and the extract was well tolerated in all dose groups. No significant changes were noted in either body weights, food consumption, or food efficiency in either sex, with the exception of a sporadic increase in food efficiency of high-dose males from days 57–64. It is recognized that a number of parameters are considered to establish whether a finding is adverse, including dose-response relationship, precision of a measurement, range of natural variation (controls), biological plausibility, magnitude of the effect, statistical significance, and whether the effects are adaptive or transient [9, 10]. Thus, statistical significance is considered when determining if a finding is toxicologically relevant, but it is not the only factor involved in this decision. Some statistically significant differences in a few hematology parameters in mid-dose and high-dose females in the recovery groups were observed, none of which were attributed to the test material because there was no effect on these parameters in main study animals. None of the statistically significant changes in clinical chemistry parameters (decreased BILI in high-dose males, decreased sodium in mid-dose males, increased HDL and ALB in all groups of treated females, increased ALB in mid-dose recovery group females, and decreased TSH in mid-dose and high-dose females) are toxicologically relevant. An increase in BILI, rather than a decrease, is reflective of toxicity to the liver. There was no effect of the test substance on total or LDL cholesterol and an increase in HDL is thought of as a beneficial effect, rather than an adverse effect [11]. Each female in all treated groups exhibited an ALB level that was within the normal range of historical laboratory control values (2.6–6.4 g/dL); therefore, the increase in mean serum albumin in all groups of treated females is not considered toxicologically relevant. For mid-dose males, the mean sodium values were also similar to historical controls (137–147 mmol/L); therefore, the result for mid-dose males is not toxicologically relevant. Decreased TSH concentration in mid-dose and high-dose females was considered an incidental change of no toxicological significance as it was not associated with any microscopic changes in the thyroid or pituitary glands of high-dose animals, and there were no effects on T3 or T4. TSH values for mid-dose and high-dose females were within limits of historical laboratory controls (1.400–10.628 ng/mL) except for one mid-dose female, who had a TSH value of 1.37 ng/mL. All groups of females (including controls) tended to have TSH values on the lower end of normal, which may have affected the results. All abnormal macroscopic or microscopic observations were incidental and not related to test material exposure as they are commonly found in Sprague–Dawley rats [12], sporadic, or also found in the controls.

There was a dose-dependent increase in liver weights in both sexes which was not observed at the end of the recovery period. While there was some hepatocellular hypertrophy in a small number of mid-dose and high-dose males, none was seen in the females. When evaluating the results of a toxicity study, it is important to interpret the data as a whole to determine if the changes in organs are adverse, adaptive, or incidental [10]. Hall et al. [13] have proposed that hepatomegaly, due to hepatocellular hypertrophy, without histological or clinical pathological evidence of liver toxicity, should be considered an adaptive and nonadverse change. Maronpot et al. [14] state that hepatocellular hypertrophy refers to an increased size of hepatocytes, not an increase in hepatocytes, and that this increase in size can be associated with a number of things including increased protein synthesis, increased cytoplasmic organelles, and/or an accumulation of intracellular components. Maronpot et al. [14] also state that hepatocellular hypertrophy is considered to be a “hallmark of enzyme induction.” There is a significant amount of information in the published literature which discusses the metabolism of cannabinoids by cytochrome P450 enzymes and the potential for cannabinoids to upregulate them [15–17]. In a review by Ennulat et al. [18], which summarized the available literature information on the effects that the induction of hepatic drug-metabolizing enzymes can have on clinical pathology parameters, the authors state that centrilobular hepatocellular hypertrophy is the most common microscopic change that is associated with enzyme induction in preclinical species. The authors also state that this effect is adaptive and does not indicate injury to the liver [18]. The evaluation of clinical pathology parameters is important in the evaluation of the effects of the phytochemicals on the liver. An increase in serum ALT or AST is seen following hepatocellular necrosis or irreversible hepatocellular injury [18]. ALKP is not liver specific in rats but its upregulation has been associated with hepatobiliary changes [18]. In rodents, SDH is reported to be a specific indicator of acute hepatocellular injury [19]. AST, ALT, ALKP, and SDH were evaluated in the reported study, and no significant differences were reported in the treated animals, as compared to concurrent controls.

Considering the lack of statistically significant changes in the liver-related clinical pathology parameters (AST, ALT, ALKP, and SDH), the mild nature of the histopathological changes in the treated animals, the absence of histopathological changes in the livers of recovery animals, and no evidence of hepatocellular degeneration, necrosis, or proliferation, it can be concluded that, under the conditions of the study, Elixinol Hemp Extract did not have adverse effects on the liver. The NOAEL for Elixinol Hemp Extract in the 90-day repeat dose study was determined to be 86.81 mg/kg bw/day for male and female Sprague–Dawley rats, which corresponds to 56.84 mg/kg bw/day CBD based on 65.48% CBD by weight in the test substance.

Elixinol Hemp Extract is a unique combination of hemp extract, CBD, and copaiba oil and given the wide variation of hemp extract compositions reported in the literature, it is difficult to make direct comparisons of values such as NOAELs across the information in published manuscripts. However, some general comparisons can be made, in particular, with the liver-related changes reported in other studies with hemp extracts. Marx et al. [6] report the results from a battery of toxicology studies for a supercritical CO_2_ extract manufactured from the aerial parts of the *C. sativa* plant. This extract was comprised of 61% edible fatty acids and 26% phytocannabinoids of which approximately 96% is CBD and less than 1% is THC and the remaining 13% is a combination of fatty alkanes, plant sterols, triterpenes, and tocopherols. Marx et al. [6] reported an increase in liver weights as well as elevated GGT levels (another enzyme-associated hepatobiliary changes [16]) but no related histopathological changes and considered these to be reversible, nonadverse effects related to exposure to the hemp extract. The authors concluded that the extract was not genotoxic and the NOAEL in a 90-day repeat dose study was 100 and 360 mg/kg bw/day in male and female Wistar rats, respectively.

Dziwenka et al. [5] reported the results of toxicological safety studies for another proprietary hemp extract which was 9% hemp extract and 91% olive oil. The hemp extract consisted of 88.70% fatty acids, 6.96% phytocannabinoids (of which 6.27% was CBD), and the final 4.34% was fatty alkanes, sterols, terpenes, and tocopherols. A bacterial reverse mutation (Ames) assay as well as *in vivo* 14-day and 90-day repeat dose toxicity studies in Sprague–Dawley rats were conducted. The authors reported increased liver weight and hepatocellular hypertrophy which was not present following the 28-day recovery period. The authors concluded that the changes were related to test material exposure but that they were reversible and not adverse. Furthermore, the extract was not mutagenic in the Ames assay and the NOAEL in the 90-day study was determined to be 400 and 800 mg/kg bw/day for males and females, respectively. In another series of studies on a proprietary hemp extract, Dziwenka, et al. [20] discussed the findings of a bacterial reverse mutation assay, *in vivo* mammalian micronucleus assay, a maximum tolerated dose study, and a 90-day repeated dose oral toxicity study. The hemp extract oil was determined to be nonmutagenic and did not induce the formation of micronuclei in an *in vivo* assay. The 90-day study included a 21-day recovery period, and the NOAEL was determined to be 90 mg/kg bw/day in both male and female Wistar rats which was the mid-dose used rather than the high-dose, based on a number of potential adverse findings but none related to the liver. This NOAEL is similar to the NOAEL proposed for Elixinol Hemp Extract (86.81 mg/kg bw/day). In the 90-day study reported by Dziwenka et al. [20], there were increases in liver weights reported in the high-dose (324 mg/kg bw/day) males and females as well as correlating minimal histological changes in the liver; however, there were no correlating changes in relevant clinical chemistry parameters. Furthermore, the findings showed no dose dependence and were reversible; therefore, the authors did not consider them to be adverse.

Epidiolex® is a prescription CBD product and is often discussed when evaluating hemp extracts but the authors feel that comparisons of studies on the safety of Epidiolex® would be most relevant when compared to CBD isolates, but not to more complex mixtures such as hemp extracts which contain various levels of CBD in addition to numerous other phytochemicals. Therefore, the results of the studies with Elixinol Hemp Extract are intentionally not compared with those of Epidiolex® in this study.

## 5. Conclusion

Elixinol Hemp Extract is a unique combination of hemp extract, CBD, and copaiba oil that was not mutagenic in a bacterial reverse mutation assay. The NOAEL of 86.81 mg/kg bw/day in male and female Sprague–Dawley rats was the highest dose administered in the 90-day repeat dose oral toxicity study. Given the significant variations that are possible in the composition of hemp extracts, it is important to determine the safety of these extracts on an individual basis. The results from the present studies indicate that Elixinol Hemp Extract is well tolerated in male and female Sprague–Dawley rats and this information contributes to the growing amount of safety information available for hemp extracts and specifically highlights the safety profile of Elixinol Hemp Extract.

## Figures and Tables

**Table 1:** Clinical chemistry data for the 90-day study of main study animals at termination (unless listed otherwise).

Parameter (historical control values^†^)	Control	28.94 mg/kg bw/day	50.64 mg/kg bw/day	86.81 mg/kg bw/day
*Males*				
AST (47–266) (U/L)	67.1 ± 7.6	66.0 ± 13.2	68.2 ± 7.3	64.9 ± 10.3
ALT (16–161) (U/L)	26.00 ± 7.41	26.10 ± 6.94	20.70 ± 3.23	26.80 ± 5.98
ALKP (36–143) (U/L)	69.9 ± 16.7	66.9 ± 10.8	75.4 ± 43.3	77.6 ± 18.0
BILI (0.02–0.90) (mg/dL)	0.062 ± 0.018	0.056 ± 0.026	0.040 ± 0.018	0.036 ± 0.022^*∗*^
BILI (0.02–0.90) (mg/dL)-RC	0.062 ± 0.019	0.054 ± 0.023	0.054 ± 0.013	0.036 ± 0.032
BUN (8–24) (mg/dL)	12.2 ± 1.3	11.5 ± 0.8	12.6 ± 1.8	12.2 ± 1.8
CREA (0.06–0.47) (mg/dL)	0.236 ± 0.025	0.260 ± 0.083	0.295 ± 0.103	0.270 ± 0.080
CHOL (36–163) (mg/dL)	50.7 ± 11.4	42.9 ± 8.2	53.6 ± 10.6	58.1 ± 9.6
LDL (0.1–0.5) (mmol/L)	0.221 ± 0.078	0.193 ± 0.060	0.219 ± 0.071	0.218 ± 0.070
HDL (0.5–2.6) (mmol/L)	0.847 ± 0.176	0.710 ± 0.121	0.871 ± 0.146	0.949 ± 0.182
TRIG (18–376) (mg/dL)	103.0 ± 69.2	90.5 ± 33.9	110.6 ± 42.8	127.4 ± 112.2
SDH (0.2–60.3) (U/L)	14.53 ± 4.66	22.70 ± 9.59	15.95 ± 3.86	19.49 ± 8.46
GLUC (77–365) (mg/dL)	215.3 ± 54.6	216.3 ± 52.1	221.5 ± 59.0	240.1 ± 76.3
TP (4.9–7.7) (g/dL)	5.83 ± 0.33	5.51 ± 0.33	5.69 ± 0.65	6.08 ± 0.45
ALB (2.8–4.6) (g/dL)	3.82 ± 0.27	3.58 ± 0.23	3.55 ± 0.50	3.94 ± 0.46
GLOB (1.3–3.5) (g/dL)	2.01 ± 0.16	1.93 ± 0.18	2.14 ± 0.40	2.14 ± 0.33
Ca (9.1–13.4) (mg/dL)	11.06 ± 0.51	11.21 ± 0.77	10.79 ± 1.00	11.53 ± 0.76
PHOS (4.6–11.7) (mg/dL)	8.72 ± 0.75	9.14 ± 0.98	8.84 ± 0.80	9.57 ± 1.31
Na (131–157) (mmol/L)	147.2 ± 3.5	147.9 ± 2.7	143.4 ± 3.6^*∗*^	145.0 ± 2.8
K (3.73–11.38) (mmol/L)	7.93 ± 0.93	8.29 ± 1.32	7.86 ± 1.05	8.76 ± 1.23
Cl (93.1–112.8) (mmol/L)	102.38 ± 2.39	102.67 ± 1.65	100.53 ± 2.78	101.13 ± 1.96
TSH (1.430–9.471) (ng/mL)	1.870 ± 0.337	1.700 ± 0.227	1.767 ± 0.332	1.652 ± 0.391
T3 (0.785–8.153) (ng/mL)	1.645 ± 0.195	1.742 ± 0.172	1.549 ± 0.106	1.574 ± 0.120
T4 (24.478–51.478) (ng/mL)	38.700 ± 1.787	41.084 ± 1.924	38.204 ± 2.362	38.812 ± 2.661

*Females*				
AST (42–341) (U/L)	55.2 ± 5.6	54.0 ± 5.8	57.1 ± 14.2	52.1 ± 6.1
ALT (13–182) (U/L)	19.20 ± 2.70	16.60 ± 3.98	19.10 ± 6.95	15.90 ± 2.60
ALKP (15–115) (U/L)	31.1 ± 7.8	32.7 ± 7.3	36.2 ± 15.3	31.3 ± 10.1
BILI (0.00 0 0.22) (mg/dL)	0.048 ± 0.022	0.035 ± 0.022	0.040 ± 0.032	0.035 ± 0.018
BUN (8–28) (mg/dL)	14.1 ± 3.1	13.7 ± 2.1	15.4 ± 4.0	13.8 ± 2.8
CREA (0.11–0.53) (mg/dL)	0.264 ± 0.080	0.254 ± 0.033	0.240 ± 0.024	0.260 ± 0.080
CHOL (28.0–249.0) (mg/dL)	67.0 ± 6.9	73.8 ± 12.8	79.7 ± 12.3	79.4 ± 15.0
LDL (0.10–0.38) (mmol/L)	0.119 ± 0.027	0.119 ± 0.021	0.138 ± 0.038	0.157 ± 0.066
HDL (0.59–2.71) (mmol/L)	1.383 ± 0.124	1.522 ± 0.236^*∗∗*^	1.662 ± 0.254^*∗∗*^	1.609 ± 0.257^*∗∗*^
HDL (0.59–2.71) (mmol/L)-RC	1.804 ± 0.425	1.970 ± 0.440	2.026 ± 0.494	2.172 ± 0.325
TRIG (24–934) (mg/dL)	111.1 ± 42.2	101.0 ± 70.2	76.6 ± 18.7	78.8 ± 20.5
SDH (0.5–81.1) (U/L)	9.94 ± 3.08	8.89 ± 1.99	13.14 ± 4.67	8.93 ± 4.10
GLUC (87–364) (mg/dL)	197.6 ± 37.3	208.7 ± 50.0	204.9 ± 33.5	182.1 ± 35.2
TP (5.1–9.0) (g/dL)	6.06 ± 0.35	6.36 ± 0.62	6.61 ± 0.63	6.49 ± 0.51
ALB (2.6–6.4) (g/dL)	4.32 ± 0.24	4.60 ± 0.42^*∗∗*^	4.82 ± 0.48^*∗∗*^	4.69 ± 0.42^*∗∗*^
ALB (2.6–6.4) (g/dL)-RC	4.86 ± 0.38	5.56 ± 0.42^*∗*^	5.18 ± 0.51	5.42 ± 0.23
GLOB (1.10–3.60) (g/dL)	1.74 ± 0.17	1.76 ± 0.25	1.79 ± 0.27	1.80 ± 0.17
Ca (7.7–15.5) (mg/dL)	10.89 ± 0.93	11.58 ± 0.88	11.81 ± 0.86	11.07 ± 0.65
PHOS (2.4–12.4) (mg/dL)	7.61 ± 1.77	8.15 ± 1.60	8.58 ± 0.70	8.03 ± 1.44
Na (128–159) (mmol/L)	144.8 ± 2.3	144.1 ± 2.3	142.5 ± 3.3	143.5 ± 3.8
K (3.49–12.96) (mmol/L)	7.24 ± 2.19	7.68 ± 1.47	8.09 ± 1.84	8.36 ± 2.35
Cl (89.1–114.6) (mmol/L)	101.93 ± 1.76	101.09 ± 2.55	99.32 ± 2.24	100.97 ± 1.85
TSH (1.400–10.628) (ng/mL)	1.731 ± 0.177	1.546 ± 0.155	1.470 ± 0.058^*∗∗∗*^	1.488 ± 0.143^+^
T3 (1.004–8.009) (ng/mL)	2.200 ± 0.436	2.571 ± 0.209	1.895 ± 0.168	1.922 ± 0.104
T4 (28.955–69.296) (ng/mL)	42.325 ± 4.025	39.696 ± 3.040	39.064 ± 2.862	38.358 ± 3.847

*N* = 10/group for the main study and *N* = 5/group for recovery groups. Data are presented as mean ± standard deviation (SD). ^†^Laboratory historical control ranges; ^*∗*^Significantly different from control, Dunnett's, or Dunn's test (*p* < 0.05); ^*∗∗*^Significantly different from control and Dunnett's test(*p* < 0.0001); ^*∗∗∗*^Significantly different from control and unknown test (*p* < 0.01); ^+^Significantly different from control and undisclosed test (*p* < 0.05). ALB = albumin; ALKP = alkaline phosphatase; ALT = alanine aminotransferase; AST = aspartate aminotransferase; BILI = total bilirubin; BUN = urea nitrogen; bw = body weight; Ca = calcium; CHOL = cholesterol; Cl = chloride; CREA = creatinine; dL = deciliter; g = grams; GLOB = globulin; GLUC = glucose; HDL = high-density lipoprotein cholesterol; K = potassium; kg = kilogram; L = liter; LDL = low-density lipoprotein cholesterol; mg = milligrams; mL = milliliter; mmol = millimoles; Na = sodium; ng = nanograms; NI = no information; PHOS = inorganic phosphorus; RC = recovery animals; SDH = sorbitol dehydrogenase; TP = total protein; TRIG = triglycerides; TSH = thyroid-stimulating hormone; thyroxine (T4); triiodothyronine (T3); U = units.

**Table 2:** Absolute organ weights (g) and relative organ to body weights (%) of 90-day study main study animals at termination (unless listed otherwise)^†^.

Parameter	Control	28.94 mg/kg bw/day	50.64 mg/kg bw/day	86.81 mg/kg bw/day
*Males*				
Terminal body weight (g)	516.0 ± 64.9	502.1 ± 75.3	536.1 ± 46.8	522.6 ± 90.1
Adrenals (g)	0.065 ± 0.011	0.055 ± 0.009	0.059 ± 0.010	0.058 ± 0.009
Adrenals/TBW	0.125 ± 0.014	0.111 ± 0.016	0.109 ± 0.017	0.114 ± 0.028
Brain (g)	2.273 ± 0.108	2.273 ± 0.084	2.306 ± 0.105	2.277 ± 0.154
Brain/TBW	4.447 ± 0.396	4.603 ± 0.584	4.320 ± 0.277	4.432 ± 0.525
Epididymides (g)	1.455 ± 0.179	1.444 ± 0.107	1.427 ± 0.190	1.432 ± 0.137
Epididymides/TBW	2.859 ± 0.483	2.930 ± 0.458	2.667 ± 0.317	2.800 ± 0.471
Heart (g)	1.487 ± 0.146	1.484 ± 0.251	1.476 ± 0.167	1.444 ± 0.214
Heart/TBW	2.902 ± 0.287	2.962 ± 0.339	2.755 ± 0.209	2.779 ± 0.240
Kidneys (g)	3.182 ± 0.301	3.367 ± 0.410	3.343 ± 0.320	3.592 ± 0.459
Kidneys/TBW	6.203 ± 0.510	6.777 ± 0.909	6.255 ± 0.568	6.948 ± 0.759
Liver (g)	13.289 ± 2.238	14.742 ± 2.425	14.795 ± 1.418	16.503 ± 2.507^*∗∗*^
Liver (g)-RC	14.726 ± 1.818	16.160 ± 2.051	15.026 ± 1.231	12.864 ± 1.524
Liver/TBW	25.653 ± 1.454	29.507 ± 3.993^*∗*^	27.623 ± 1.632	31.796 ± 2.819^*∗∗∗*^
Liver/TBW-RC	25.851 ± 0.874	26.093 ± 1.947	26.165 ± 1.249	24.990 ± 1.546
Liver/TBrW	5.825 ± 0.765	6.466 ± 0.885	6.410 ± 0.455	7.239 ± 0.847^*∗∗∗*^
Liver/TBrW-RC	6.243 ± 0.401	6.633 ± 0.854	6.224 ± 0.733	5.776 ± 0.708
Spleen (g)	0.927 ± 0.186	0.899 ± 0.173	0.890 ± 0.155	0.943 ± 0.188
Spleen/TBW	1.784 ± 0.195	1.795 ± 0.264	1.653 ± 0.194	1.868 ± 0.608
Testes (g)	3.781 ± 0.215	3.777 ± 0.221	3.768 ± 0.419	3.753 ± 0.285
Testes/TBW	7.432 ± 1.019	7.627 ± 0.834	7.050 ± 0.761	7.342 ± 1.187
Thymus (g)	0.255 ± 0.054	0.278 ± 0.095	0.232 ± 0.070	0.276 ± 0.094
Thymus/TBW	0.497 ± 0.110	0.552 ± 0.160	0.433 ± 0.122	0.524 ± 0.145

*Females*				
Terminal body weight (g)	317.2 ± 34.3	304.7 ± 33.2	296.1 ± 21.1	297.6 ± 20.2
Adrenals (g)	0.081 ± 0.014	0.080 ± 0.009	0.080 ± 0.008	0.077 ± 0.016
Adrenals/TBW	0.257 ± 0.045	0.266 ± 0.032	0.270 ± 0.032	0.258 ± 0.051
Brain (g)	2.152 ± 0.058	2.157 ± 0.080	2.135 ± 0.136	2.142 ± 0.108
Brain/TBW	6.855 ± 0.752	7.140 ± 0.665	7.226 ± 0.450	7.219 ± 0.471
Heart (g)	1.053 ± 0.133	1.061 ± 0.120	1.008 ± 0.083	1.002 ± 0.055
Heart/TBW	3.320 ± 0.200	3.491 ± 0.287	3.414 ± 0.304	3.375 ± 0.217
Kidneys (g)	2.158 ± 0.219	2.171 ± 0.223	2.145 ± 0.215	2.255 ± 0.253
Kidneys/TBW	6.818 ± 0.383	7.154 ± 0.615	7.241 ± 0.486	7.594 ± 0.867
Liver (g)	9.327 ± 1.180	9.347 ± 1.467	9.804 ± 1.184	9.958 ± 0.696
Liver (g)-RC	8.328 ± 1.267	9.428 ± 0.846	10.020 ± 1.921	10.096 ± 0.752
Liver/TBW	29.360 ± 0.982	30.588 ± 2.199	33.120 ± 3.326^*∗∗*^	33.511 ± 2.089^*∗∗∗*^
Liver/TBW-RC	27.438 ± 2.664	30.158 ± 3.064	29.970 ± 3.019	31.238 ± 1.738
Liver/TBrW	4.336 ± 0.557	4.324 ± 0.577	4.593 ± 0.477	4.654 ± 0.330
Liver/TBrW-RC	3.926 ± 0.664	4.429 ± 0.534	4.693 ± 0.772	4.737 ± 0.435
Ovaries (g)	0.134 ± 0.024	0.135 ± 0.020	0.141 ± 0.014	0.140 ± 0.018
Ovaries/TBW	0.424 ± 0.070	0.447 ± 0.068	0.477 ± 0.040	0.470 ± 0.057
Spleen (g)	0.582 ± 0.084	0.621 ± 0.081	0.589 ± 0.077	0.658 ± 0.056
Spleen/TBW	1.835 ± 0.190	2.042 ± 0.213	1.987 ± 0.183	2.216 ± 0.189^*∗∗∗*^
Thymus (g)	0.274 ± 0.069	0.257 ± 0.098	0.306 ± 0.096	0.270 ± 0.041
Thymus/TBW	0.862 ± 0.176	0.842 ± 0.300	1.035 ± 0.325	0.913 ± 0.162
Uterus (g)	0.794 ± 0.218	0.881 ± 0.311	0.844 ± 0.257	0.807 ± 0.211
Uterus/TBW	2.517 ± 0.723	2.873 ± 0.917	2.898 ± 1.077	2.703 ± 0.631

*N* = 10/group for the main study and *N* = 5/group for recovery groups. Data are presented as mean ± standard deviation (SD). Relative organ weights (ratios) presented in the table are times 1000. ^†^Data for organ weight as a % of brain weight, as well as data for recovery group animals are included in the table if absolute organ weight or organ weight as a % of body weight in main study animals was affected. ^*∗*^Significantly different from control by Dunnett's or Dunn's test (2-sided) (*p* < 0.05); ^*∗∗*^Significantly different from control by Dunnett's or Dunn's test (2-sided) (*p* < 0.01); ^*∗∗∗*^Significantly different from control by Dunnett's or Dunn's test (2-sided) (*p* < 0.001). bw = body weight; g = grams; kg = kilogram; mg = milligrams; RC = recovery group; TBW = terminal body weight; TBrW = terminal brain weight.

## Data Availability

The data not supplied in the publication can be found in the supplementary material.

## Supplementary Materials

Supplementary Materials Supplementary Table 1: mean number of revertants/plate for the reverse mutation assay of Elixinol Hemp Extract in *Salmonella typhimurium* and *Escherichia coli*; Supplementary Table 2: mean body weight gain of male and female rats during the 90-day study; Supplementary Table 3: mean food consumption of male and female rats during the 90-day study; Supplementary Table 4: hematology and coagulation data for the 90-day main study animals at termination; Supplementary Table 5: histopathology summary for Elixinol Hemp Extract.
