# Supplementary material for: Safety of Elixinol Hemp Extract: In Vitro Genetic Toxicity and Subchronic Toxicity in Rats
Source: J Toxicol. 2023 Dec 11;2023:5982883. doi: 10.1155/2023/5982883 (PMC10727801; doi:10.1155/2023/5982883)
Supplement: Supplementary Materials — Supplementary Table 1: mean number of revertants/plate for the reverse mutation assay of Elixinol Hemp Extract in Salmonella typhimurium and Escherichia coli; Supplementary Table 2: mean body weight gain of male and female rats during the 90-day study; Supplementary Table 3: mean food consumption of male and female rats during the 90-day study; Supplementary Table 4: hematology and coagulation data for the 90-day main study animals at termination; Supplementary Table 5: histopathology summary for Elixinol Hemp Extract. [file 5982883.f1.docx]

**Supplementary Table 1**. Reverse mutation assay of Elixinol Hemp Extract in *Salmonella typhimurium* and *Escherichia coli*: mean number of revertants/plate

| Concentration (µg/plate) | TA98 | | TA100 | | TA1535 | | TA1537 | | WP2*uvrA* | |
| --- | --- | --- | --- | --- | --- | --- | --- | --- | --- | --- |
|  | -S9 | +S9 | -S9 | +S9 | -S9 | +S9 | -S9 | +S9 | -S9 | +S9 |
| Experiment 1 | | | | | | | | | | |
| 0^a^ | 30 | 26 | 83 | 92 | 16 | 13 | 9 | 10 | 37 | 44 |
| 2.286 | 26 | 34 | 87 | 87 | 16 | 11 | 8 | 11 | 37 | 46 |
| 7.234 | 27 | 35 | 90 | 91 | 13 | 9 | 9 | 16 | 38 | 44 |
| 22.86 | 22 | 30 | 81 | 86 | 15 | 9 | 12 | 12 | 38 | 41 |
| 72.34 | 19 | 30 | 81 | 81 | 14 | 11 | 9 | 13 | 36 | 41 |
| 228.6 | 21 | 26 | 79 | 73 | 9 | 10 | 8 | 9 | 31 | 39 |
| 723.4 | 19 | 21 | 56 | 61 | 12 | 8 | 7 | 4 | 41 | 34 |
| 2286 | 20* | 20* | 67* | 65* | 18* | 9* | 6* | 10* | 37* | 34* |
| 7234 | 16* | 19* | 64* | 67* | 13* | 10* | 6* | 6* | 35* | 39* |
| Positive control | 1300^b^ | 3707^c^ | 459^d^ | 3011^c^ | 259^d^ | 353^c^ | 305^e^ | 379^c^ | 538^f^ | 109^c^ |
| Experiment 2 |  |  |  |  |  |  |  |  |  |  |
| 0^a^ | 22 | 20 | 90 | 85 | 11 | 9 | 7 | 8 | 41 | 41 |
| 2.286 | 25 | 22 | 87 | 92 | 12 | 8 | 6 | 9 | 37 | 43 |
| 7.234 | 20 | 25 | 88 | 88 | 14 | 10 | 7 | 8 | 39 | 39 |
| 22.86 | 20 | 23 | 82 | 87 | 12 | 9 | 10 | 8 | 40 | 41 |
| 72.34 | 18 | 23 | 83 | 81 | 10 | 9 | 7 | 9 | 29 | 41 |
| 228.6 | 17 | 26 | 80 | 84 | 16 | 9 | 5 | 7 | 34 | 37 |
| 723.4 | 17 | 21 | 82** | 73 | 9 | 10 | 3 | 6 | 35 | 40 |
| 2286 | 19* | 22* | 73* | 72* | 10* | 13* | 2* | 3* | 35 | 35 |
| 7234 | 20* | 20* | 70* | 77* | 11* | 9* | 5* | 5* | 31 | 37 |
| Positive control | 984^b^ | 3484^c^ | 424^d^ | 3540^c^ | 225^d^ | 361^c^ | 3689^e^ | 396^c^ | 409^f^ | 128^c^ |

Substance was tested using the standardized plate incorporation assay (Experiment 1) and the pre-incubation method (Experiment 2). Results are means of three replicates per test condition. S9= bioactivation system.

^a^DMSO vehicle; ^b^daunomycin; ^c^2-aminoanthracene; ^d^Sodium azide; ^e^ICR 191 Acridine; ^f^MMS

*Precipitate observed; **Incomplete lawn

**Supplementary Table 2.** Mean daily body weight gain of male and female rats during the 90-day study (g/day)

| Day of Study | Control | 28.94 mg/kg bw/day | 50.64 mg/kg bw/day | 86.81 mg/kg bw/day |
| --- | --- | --- | --- | --- |
| **Males** | | | | |
| 1 → 8 | 6.45 ± 1.44 | 5.90 ± 1.46 | 7.24 ± 2.05 | 6.10 ± 1.99 |
| 8 → 15 | 7.17 ± 2.75 | 7.00 ± 2.09 | 5.62 ± 2.62 | 6.34 ± 1.95 |
| 15 → 22 | 5.18 ± 2.78 | 5.66 ± 1.51 | 6.57 ± 2.54 | 5.54 ± 1.40 |
| 22 → 29 | 5.02 ± 0.85 | 5.30 ± 1.11 | 5.14 ± 1.11 | 4.71 ± 2.01 |
| 29 → 36 | 3.70 ± 1.12 | 3.86 ± 1.19 | 3.53 ± 0.82 | 3.16 ± 0.91 |
| 36 → 43 | 3.22 ± 1.06 | 3.74 ± 1.24 | 2.96 ± 0.91 | 3.34 ± 0.86 |
| 43 → 50 | 3.00 ± 1.27 | 3.12 ± 1.18 | 3.34 ± 1.36 | 3.37 ± 0.77 |
| 50 → 57 | -1.98 ± 16.60 | 2.58 ± 0.65 | 2.48 ± 0.64 | 2.18 ± 1.04 |
| 57 → 64 | 5.93 ± 16.76 | 2.04 ± 0.78 | 1.75 ± 0.89 | 2.44 ± 0.70 |
| 64 → 71 | 2.27 ± 0.83 | 2.47 ± 1.00 | 2.34 ± 0.92 | 1.43 ± 1.27 |
| 71 → 78 | 1.59 ±­ 1.03 | 1.55 ± 1.25 | 1.85 ± 0.46 | 1.57 ± 0.95 |
| 78 → 85 | 2.21 ± 0.76 | 1.84 ± 1.08 | 2.07 ± 1.27 | 1.08 ± 2.31 |
| 85→ 92 | 1.06 ± 0.91 | 1.84 ± 1.02 | 1.82 ± 1.19 | 1.48 ± 1.99 |
| 1→ 92 | 3.45 ± 0.53 | 3.61 ± 0.71 | 3.59 ± 0.47 | 3.29 ± 0.78 |
| 92 → 99 | 0.31 ± 1.03 | 0.83 ± 1.26 | 0.80 ± 0.78 | 1.00 ± 0.85 |
| 99 → 106 | 1.29 ± 2.32 | 0.77 ± 1.69 | 2.40 ± 1.87 | 1.86 ± 1.13 |
| 106 → 113 | 2.91 ± 1.47 | 2.51 ± 1.04 | 1.71 ± 0.48 | 2.34 ± 0.67 |
| 113 → 120 | 3.63 ± 2.69 | 4.06 ± 2.90 | 4.03 ± 2.17 | 3.11 ± 2.29 |
| 92 → 120 | 2.04 ± 0.74 | 2.04 ± 0.47 | 2.24 ± 0.50 | 2.08 ± 0.62 |
| 1 → 120 | 3.11 ± 0.41 | 3.49 ± 0.45 | 3.22 ± 0.49 | 2.73 ± 0.37 |
| **Females** | | | | |
| 1 → 8 | 5.40 ± 1.01 | 4.98 ± 1.00 | 4.45 ± 0.96 | 4.47 ± 0.63 |
| 8 → 15 | 3.41 ± 0.97 | 3.29 ± 1.03 | 3.05 ± 1.82 | 3.57 ± 0.92 |
| 15 → 22 | 3.25 ± 0.79 | 3.10 ± 1.33 | 3.19 ± 1.57 | 3.13 ± 1.01 |
| 22 → 29 | 1.98 ± 0.70 | 2.36 ± 1.43 | 2.00 ± 1.11 | 2.34 ± 0.78 |
| 29 → 36 | 1.94 ± 2.88 | 1.88 ± 0.97 | 1.92 ± 0.97 | 1.86 ± 0.89 |
| 36 → 43 | 1.66 ± 3.09 | 1.10 ± 0.93 | 1.47 ± 0.82 | 1.60 ± 0.65 |
| 43 → 50 | 1.28 ± 1.04 | 1.48 ± 0.98 | 1.25 ± 0.79 | 1.29 ± 0.57 |
| 50 → 57 | 0.96 ± 0.70 | 0.95 ± 0.82 | 0.98 ± 0.85 | 1.19 ± 0.87 |
| 57 → 64 | 1.21 ± 1.16 | 0.87 ± 0.71 | 1.45 ± 0.96 | 1.38 ± 0.79 |
| 64 → 71 | 0.75 ± 0.70 | 0.96 ± 0.68 | 0.53 ± 0.81 | 0.53 ± 0.57 |
| 71 → 78 | 0.74 ± 0.84 | 0.67 ± 0.63 | 0.61 ± 2.77 | 0.07 ± 0.80 |
| 78 → 85 | 1.09 ± 1.10 | 0.78 ± 1.08 | 1.12 ± 2.49 | 1.13 ± 0.98 |
| 85→ 92 | 0.61 ± 0.87 | 0.40 ± 0.83 | -0.18 ± 2.44 | 0.93 ± 1.22 |
| 1→ 92 | 1.87 ± 0.28 | 1.76 ± 0.26 | 1.68 ± 0.18 | 1.81 ± 0.22 |
| 92 → 99 | 0.11 ± 0.91 | 0.74 ± 0.77 | 3.46 ± 4.70 | -0.57 ± 0.78 |
| 99 → 106 | 0.89 ± 0.58 | 1.80 ± 0.73 | 0.89 ± 0.93 | 1.29 ± 0.40 |
| 106 → 113 | 0.54 ± 0.99 | 0.89 ± 1.14 | 1.89 ± 1.59 | 1.06 ± 0.85 |
| 113 → 120 | 0.23 ± 0.44 | 0.60 ± 0.67 | 1.80 ± 2.88 | 0.83 ± 0.51 |
| 92 → 120 | 0.44 ± 0.48 | 1.01 ± 0.47 | 2.01 ± 1.89 | 0.65 ± 0.22 |
| 1 → 120 | 1.43 ± 0.12 | 1.56 ± 0.25 | 1.65 ± 0.43 | 1.61 ± 0.28 |

**Supplementary Table 3.** Mean food consumption of male and female rats during the 90-day study

| Day of Study | Control | 28.94 mg/kg bw/day | 50.64 mg/kg bw/day | 86.81 mg/kg bw/day |
| --- | --- | --- | --- | --- |
| **Males** | | | | |
| 1 → 8 | 20.77 ± 1.29 | 20.12 ± 2.58 | 21.81 ± 2.59 | 20.47 ± 2.21 |
| 8 → 15 | 23.26 ± 1.81 | 22.97 ± 2.80 | 23.71 ± 2.51 | 23.48 ± 2.78 |
| 15 → 22 | 22.99 ± 1.35 | 23.37 ± 2.69 | 24.79 ± 3.17 | 22.93 ± 3.08 |
| 22 → 29 | 23.64 ± 1.86 | 23.23 ± 2.51 | 25.19 ± 4.89 | 22.71 ± 3.01 |
| 29 → 36 | 22.98 ± 1.04 | 23.01 ± 2.96 | 23.12 ± 1.62 | 21.97 ± 2.63 |
| 36 → 43 | 24.87 ± 1.74 | 24.73 ± 2.33 | 23.04 ± 1.88* | 22.20 ± 2.39** |
| 43 → 50 | 24.80 ± 1.67 | 24.60 ± 2.93 | 24.26 ± 1.47 | 23.50 ± 2.22 |
| 50 → 57 | 22.35 ± 1.18 | 22.72 ± 1.80 | 22.80 ± 0.96 | 21.70 ± 2.76 |
| 57 → 64 | 23.09 ± 1.57 | 22.48 ± 1.94 | 22.55 ± 1.24 | 22.06 ± 2.47 |
| 64 → 71 | 22.30 ± 2.27 | 22.50 ± 2.09 | 23.30 ± 1.87 | 21.70 ± 2.13 |
| 71 → 78 | 25.89 ± 2.97 | 23.86 ± 2.54 | 23.37 ± 1.21* | 20.73 ± 3.45** |
| 78 → 85 | 23.49 ± 2.34 | 20.82 ± 2.91* | 21.90 ± 1.15 | 19.45 ± 3.09** |
| 85→ 92 | 21.40 ± 3.27 | 19.66 ± 2.78 | 19.04 ± 2.52 | 20.50 ± 2.85 |
| 1→ 92 | 23.22 ± 1.35 | 22.62 ± 2.21 | 22.99 ± 1.55 | 21.80 ± 2.41 |
| 92 → 99 | 25.51 ± 0.97 | 26.71 ± 3.00 | 26.34 ± 1.51 | 24.17 ± 0.81 |
| 99 → 106 | 28.63 ± 2.23 | 29.51 ± 2.79 | 30.37 ± 0.25 | 27.46 ± 0.22 |
| 106 → 113 | 30.37 ± 1.88 | 30.69 ± 3.09 | 28.86 ± 0.07 | 25.57 ± 4.24 |
| 113 → 120 | 32.43 ± 1.70 | 33.91 ± 0.97 | 28.40 ± 4.47 | 29.40 ± 2.97 |
| 92 → 120 | 29.24 ± 0.84 | 30.21 ± 1.98 | 28.49 ± 0.69 | 26.65 ± 2.06* |
| 1 → 120 | 24.98 ± 0.51 | 24.82 ± 2.36 | 23.34 ± 0.39 | 21.79 ± 0.71** |
| **Females** | | | | |
| 1 → 8 | 16.09 ± 1.26 | 15.89 ± 0.84 | 15.42 ± 1.24 | 15.30 ± 0.61 |
| 8 → 15 | 16.77 ± 1.50 | 16.20 ± 0.97 | 16.16 ± 1.56 | 15.80 ± 0.75 |
| 15 → 22 | 18.30 ± 1.32 | 16.41 ± 2.58* | 18.08 ± 1.29 | 18.37 ± 1.37 |
| 22 → 29 | 17.54 ± 1.36 | 17.24 ± 0.98 | 17.53 ± 1.89 | 17.76 ± 1.24 |
| 29 → 36 | 18.20 ± 1.64 | 18.06 ± 1.61 | 18.10 ± 1.85 | 17.72 ± 2.77 |
| 36 → 43 | 19.42 ± 1.74 | 18.50 ± 1.44 | 18.25 ± 2.02 | 17.79 ± 1.07 |
| 43 → 50 | 19.30 ± 2.16 | 18.54 ± 1.22 | 18.93 ± 2.18 | 18.07 ± 1.48 |
| 50 → 57 | 17.58 ± 1.92 | 17.59 ± 0.94 | 18.13 ± 2.13 | 17.81 ± 1.37 |
| 57 → 64 | 17.75 ± 1.64 | 17.54 ± 1.52 | 17.51 ± 2.31 | 17.36 ± 1.14 |
| 64 → 71 | 16.90 ± 1.64 | 16.19 ± 3.68 | 17.21 ± 2.04 | 17.08 ± 1.59 |
| 71 → 78 | 19.18 ± 1.87 | 18.71 ± 1.37 | 18.01 ± 2.45 | 17.46 ± 1.81* |
| 78 → 85 | 16.99 ± 2.10 | 15.59 ± 1.31 | 16.40 ± 2.33 | 15.96 ± 1.89 |
| 85→ 92 | 16.75 ± 4.02 | 12.43 ± 3.08 | 16.45 ± 3.10 | 15.50 ± 1.13 |
| 1→ 92 | 17.75 ± 1.62 | 16.84 ± 1.36 | 17.40 ± 1.85 | 17.07 ± 1.27 |
| 92 → 99 | 17.54 ± 0.81 | 19.37 ± 0.14** | 21.26 ± 1.15** | 18.86 ± 1.17** |
| 99 → 106 | 18.89 ± 0.95 | 20.63 ± 1.32* | 22.43 ± 2.87* | 22.31 ± 0.30** |
| 106 → 113 | 18.54 ± 1.46 | 21.31 ± 1.67** | 23.31 ± 2.50** | 23.37 ± 0.18** |
| 113 → 120 | 18.17 ± 1.80 | 21.09 ± 1.68** | 23.49 ± 0.97** | 21.80 ± 0.01** |
| 92 → 120 | 18.29 ± 1.26 | 20.60 ± 1.13** | 22.62 ± 1.87** | 21.59 ± 0.41** |
| 1 → 120 | 17.10 ± 0.54 | 16.73 ± 1.14 | 18.88 ± 2.19 | 18.75 ± 0.05 |

N = 15/group from Days 1 to 92 and 5/group from days 99-120. Data are presented as mean ± standard deviation (SD). *Significantly different from control, 2 Way ANOVA, *p*<0.05; **Significantly different from control, 2 Way ANOVA, *p*<0.01. bw = body weight; kg = kilogram; mg = milligrams.

**Supplementary Table 4**. Hematology and Coagulation Data for the 90-Day Main Study Animals at Termination

| Parameter (historical control values^†^) | Control | 28.94 mg/kg  bw/day | 50.64 mg/kg  bw/day | 86.81 mg/kg  bw/day |
| --- | --- | --- | --- | --- |
| **Males** | | | | |
| RBC (7.73 – 10.08) (10^6^/µL) | 8.765 ± 0.383 | 8.574 ± 0.345 | 8.704 ± 0.277 | 8.450 ± 0.448 |
| HGB (13.7 – 19.1) (g/dL) | 15.51 ± 0.39 | 14.95 ± 0.51 | 15.10 ± 0.42 | 14.82 ± 1.01 |
| HCT (43.9 – 55.3) (%) | 51.32 ± 1.57 | 49.41 ± 1.59 | 49.81 ± 1.53 | 49.05 ± 2.77 |
| MCV (50.4 – 62.4) (fL) | 58.60 ± 1.43 | 57.67 ± 1.82 | 57.23 ± 1.63 | 58.12 ± 2.96 |
| MCH (15.4 – 21.6) (pg) | 17.73 ± 0.50 | 17.44 ± 0.61 | 17.37 ± 0.52 | 17.53 ± 1.00 |
| MCHC (29.9 – 36.9) (g/dL) | 30.2 ± 0.4 | 30.3 ± 0.4 | 30.3 ± 0.3 | 30.4 ± 0.3 |
| RDW (10.9 – 17.2) (%) | 13.10 ± 0.49 | 13.22 ± 0.65 | 13.07 ± 0.36 | 13.08 ± 0.92 |
| RET (43.90 – 414.7) (10^3^/µL) | 169.8 ± 20.3 | 166.7 ± 31.3 | 154.4 ± 30.9 | 157.6 ± 48.7 |
| PLT (650 – 1517) (10^3^/µL) | 1105.0 ± 148.7 | 1115.2 ± 67.6 | 1145.7 ± 122.5 | 1112.4 ± 227.2 |
| WBC (5.66 – 24.27) (10^3^/µL) | 10.688 ± 2.415 | 12.089 ± 3.483 | 10.488 ± 2.225 | 11.616 ± 2.393 |
| NEU (0.65 – 8.79) (10^3^/µL) | 1.551 ± 1.404 | 1.739 ± 0.534 | 1.656 ± 0.654 | 1.992 ± 1.270 |
| LYM (4.17 – 20.00) (10^3^/µL) | 8.520 ± 2.0357 | 9.654 ± 3.101 | 8.175 ± 1.919 | 8.960 ± 1.640 |
| MON (0.10 – 0.83) (10^3^/µL) | 0.30 ± 0.07 | 0.37 ± 0.15 | 0.34 ± 0.10 | 0.31 ± 0.04 |
| EOS (0.04 – 0.64) (10^3^/µL) | 0.094 ± 0.028 | 0.091 ± 0.028 | 0.098 ± 0.042 | 0.083 ± 0.034 |
| BAS (0.01 – 0.32) (10^3^/µL) | 0.063 ± 0.032 | 0.050 ± 0.019 | 0.060 ± 0.037 | 0.060 ± 0.029 |
| LUC (0.01 – 0.50) (10^3^/µL) | 0.156 ± 0.056 | 0.187 ± 0.120 | 0.168 ± 0.080 | 0.216 ± 0.090 |
| APTT (14.1 – 37.6) (s) | 17.6 ± 3.1 | 18.2 ± 5.8 | 16.9 ± 2.0 | 18.1 ± 3.0 |
| PT (8.5 – 10.7) (s) | 9.7 ± 0.2 | 9.9 ± 0.3 | 9.8 ± 0.3 | 9.7 ± 0.3 |
| **Females** | | | | |
| RBC (6.74 – 9.74) (10^6^/µL) | 8.012 ± 0.383 | 8.062 ± 0.327 | 8.014 ± 0.348 | 8.120 ± 0.259 |
| HGB (12.5 – 17.8) (g/dL) | 14.79 ± 0.33 | 14.72 ± 0.47 | 14.70 ± 0.46 | 14.98 ± 0.39 |
| HCT (39.4 – 52.0) (%) | 47.23 ± 1.65 | 47.07 ± 1.97 | 46.96 ± 1.56 | 47.91 ± 1.60 |
| MCV (51.2 – 61.5) (fL) | 58.99 ± 1.13 | 58.36 ± 1.21 | 58.64 ± 1.86 | 59.02 ± 1.86 |
| MCH (16.4 – 21.3) (pg) | 18.48 ± 0.57 | 18.26 ± 0.48 | 18.36 ± 0.74 | 18.46 ± 0.63 |
| MCHC (30.6 – 36.1) (g/dL) | 31.3 ± 0.5 | 31.3 ± 0.5 | 31.3 ± 0.4 | 31.3 ± 0.6 |
| RDW (10.1 – 15.8) (%) | 11.52 ± 0.36 | 11.53 ± 0.37 | 11.51 ± 0.22 | 11.48 ± 0.31 |
| RET (2.30 – 542.70) (10^3^/µL) | 135.3 ± 18.5 | 134.4 ± 39.7 | 122.3 ± 23.8 | 147.2 ± 26.6 |
| PLT (698.0 – 1641.0) (10^3^/µL) | 1027.1 ± 156.3 | 1010.3 ± 99.4 | 968.4 ± 116.9 | 994.8 ± 119.2 |
| WBC (2.75 – 17.16) (10^3^/µL) | 5.924 ± 1.454 | 6.109 ± 1.474 | 7.552 ± 2.354 | 7.400 ± 1.828 |
| NEU (0.31 – 7.26) (10^3^/µL) | 0.892 ± 0.427 | 0.935 ± 0.416 | 0.914 ± 0.309 | 1.005 ± 0.457 |
| LYM (2.1 – 12.09) (10^3^/µL) | 4.705 ± 1.220 | 4.818 ± 1.219 | 6.228 ± 2.027 | 6.011 ± 1.702 |
| MON (0.04 – 0.81) (10^3^/µL) | 0.17 ± 0.04 | 0.18 ± 0.09 | 0.20 ± 0.06 | 0.17 ± 0.04 |
| EOS (0.03 – 0.34) (10^3^/µL) | 0.043 ± 0.012 | 0.058 ± 0.024 | 0.059 ± 0.034 | 0.074 ± 0.046 |
| BAS (0.00 – 0.23) (10^3^/µL) | 0.027 ± 0.015 | 0.029 ± 0.014 | 0.037 ± 0.016 | 0.039 ± 0.019 |
| LUC (0.01 – 0.31) (10^3^/µL) | 0.086 ± 0.038 | 0.084 ± 0.044 | 0.113 ± 0.034 | 0.101 ± 0.037 |
| APTT (11.2 – 30.5) (s) | 16.1 ± 1.6 | 16.4 ± 1.4 | 17.0 ± 2.9 | 16.9 ± 1.2 |
| PT (8.3 – 11.0) (s) | 9.5 ± 0.2 | 9.6 ± 0.3 | 9.4 ± 0.3 | 9.5 ± 0.2 |

N = 10/group. Data are presented as mean ± standard deviation (SD). There are no statistically significant differences between groups at *p*<0.05. ^†^Laboratory historical control ranges; APTT = activated partial thromboplastin time; BAS = basophils; bw = body weight; dL = deciliter; EOS = eosinophils; fL = femtoliter; HCT = hematocrit; HGB = hemoglobin; kg = kilogram; LUC = large unstained cells; LYM = lymphocytes; MCH = mean corpuscular hemoglobin; MCHC = mean corpuscular hemoglobin concentration; MCV = mean corpuscular volume; mg = milligrams; MON = monocytes; NEU = neutrophils; pg = picograms; PLT = platelets; PT = prothrombin time; RBC = erythrocytes; RDW = red blood cell distribution width; RET = reticulocytes; s = seconds; WBC = white blood cells (leukocytes); µL = microliter.

**Supplementary Table 5.** Histopathology Summary for Elixinol Hemp Extract

|  | **Main Study** | | | | **Recovery** | | | |
| --- | --- | --- | --- | --- | --- | --- | --- | --- |
|  | **Control**  **(n=10)** | **28.94 mg/kg**  **(n=10)** | **50.64 mg/kg**  **(n=10)** | **86.81 mg/kg**  **(n=10)** | **Control**  **(n=5)** | **28.94 mg/kg**  **(n=5)** | **50.64 mg/kg**  **(n=5)** | **86.81 mg/kg**  **(n=5)** |
| **Males** | | | | | | | | |
| Adrenal gland – vacuolation | 0 | NE | NE | 1 (minimal)  1 (mild) | NE | NE | NE | NE |
| Bone Marrow – granulocytic hyperplasia | 0 | NE | NE | 1 (moderate) | NE | NE | NE | NE |
| Esophagus – fibrosis | 0 | NE | NE | 1 (moderate) | NE | NE | NE | NE |
| Esophagus – inflammation | 0 | NE | NE | 1 (mild) | NE | NE | NE | NE |
| Harderian gland - infiltrate | 1 (mild) | NE | NE | 0 | NE | NE | NE | NE |
| Heart - fibrosis | 1 (minimal) | NE | NE | 0 | NE | NE | NE | NE |
| Heart - infiltrate | 1 (minimal) | NE | NE | 0 | NE | NE | NE | NE |
| Kidney – tubular basophilia | 1 (minimal) | NE | NE | 1 (minimal) | NE | NE | NE | NE |
| Kidney – cyst | 3 (present) | NE | NE | 0 | NE | NE | NE | NE |
| Kidney – dilation | 0 | NE | NE | 2 (mild) | NE | NE | NE | NE |
| Kidney – urothelium hyperplasia | 0 | NE | NE | 1 (moderate) | NE | NE | NE | NE |
| Kidney - infiltrate | 1 (minimal) | NE | NE | 1 (minimal) | NE | NE | NE | NE |
| Kidney - inflammation | 0 | NE | NE | 1 (minimal) | NE | NE | NE | NE |
| Larynx – inflammation | 1 (minimal) | NE | NE | 1 (marked) | NE | NE | NE | NE |
| Liver – hepatocellular hyperplasia | 0 | 0 | 2 (minimal) | 2 (minimal) | 0 | 0 | 0 | 0 |
| Liver – hepatocellular vacuolation | 0 | 0 | 0 | 1 (minimal) | 0 | 0 | 0 | 0 |
| Lungs – macrophage accumulation | 3 (minimal) | NE | NE | 1 (minimal) | NE | NE | NE | NE |
| Lungs - infiltrate | 4 (minimal)  2 (mild) | NE | NE | 2 (minimal) | NE | NE | NE | NE |
| Pancreas – focal atrophy/fibrosis | 1 (minimal) | 2 (mild) | 2 (mild) | 0 | 1 (minimal) | 0 | 0 | 0 |
| Pancreas – islet cell fibrosis | 0 | 1 (minimal) | 2 (mild) | 0 | 0 | 0 | 1 (minimal) | 0 |
| Pancreas - infiltrate | 0 | 0 | 0 | 1 (minimal) | 0 | 0 | 0 | 0 |
| Prostate – cyst | 0 | NE | NE | 1 (present) | NE | NE | NE | NE |
| Stomach – mucosal dilation | 3 (minimal) | NE | NE | 1 (minimal) | NE | NE | NE | NE |
| Testes – degeneration/atrophy | 0 | NE | NE | 2 (minimal) | NE | NE | NE | NE |
| Thymus – congestion | 0 | NE | 1 (minimal) | 0 | NE | NE | NE | NE |
| Urinary bladder – hyperplasia | 0 | NE | NE | 1 (marked) | NE | NE | NE | NE |
| Urinary bladder - mineralization | 0 | NE | NE | 1 (moderate) | NE | NE | NE | NE |
| **Females** | | | | | | | | |
| Esophagus – fibrosis | 1 (mild) | NE | NE | 0 | NE | NE | NE | NE |
| Harderian gland - infiltrate | 1 (mild) | NE | NE | 0 | NE | NE | NE | NE |
| Kidney – cyst | 0 | NE | NE | 1 (present) | NE | NE | NE | NE |
| Kidney - infiltrate | 2 (minimal) | NE | NE | 0 | NE | NE | NE | NE |
| Lungs – macrophage accumulation | 0 | NE | NE | 1 (minimal) | NE | NE | NE | NE |
| Lungs - infiltrate | 1 (minimal) | NE | NE | 1 (mild) | NE | NE | NE | NE |
| Pituitary gland – aberrant craniopharngeal structures | 1 (minimal) | NE | NE | 0 | NE | NE | NE | NE |
| Pancreas – focal atrophy/fibrosis | 1 (mild) | NE | NE | 1 (minimal) | NE | NE | NE | NE |
| Stomach – mucosal dilation | 1 (minimal) | NE | NE | 3 (minimal)  1 (mild) | NE | NE | NE | NE |
| Uterus - dilation | 2 (minimal)  1 (mild) | 1 (minimal)  1 (moderate) | 1 (mild) | 1 (minimal)  2 (mild) | NE | NE | NE | NE |
| NE – not examined | | | | | | | | |
